# Significant Impacts of Work-Related Cerebrovascular and Cardiovascular Diseases among Young Workers: A Nationwide Analysis

**DOI:** 10.3390/ijerph16060961

**Published:** 2019-03-18

**Authors:** Ya-Yuan Hsu, Ray Wang, Chyi-Huey Bai

**Affiliations:** 1 Division of Labor Market, Institute of Labor, Occupational Safety, and Health, Ministry of Labor, Taipei 221, Taiwan; yayuarn@mail.ilosh.gov.tw; 2 School of Public Health, College of Public Health, Taipei Medical University, Taipei 110, Taiwan; 3 Institute of Epidemiology and Preventive Medicine, College of Public Health, National Taiwan University, Taipei 100, Taiwan; romanray92@gmail.com; 4 Department of Public Health, College of Medicine, Taipei Medical University, Taipei 110, Taiwan; 5 Research Center of Health Equity, College of Public Health, Taipei Medical University, Taipei 110, Taiwan

**Keywords:** work-related cerebrovascular and cardiovascular diseases, occupation, Poisson regression, rate ratio

## Abstract

*Background*: While occupational factors linked to the onset of cerebrovascular and cardiovascular diseases (CVDs) have been reported among workers, much remains unknown about the impacts that occupation has on the onset of CVDs in various age groups. We attempted to describe temporal trends in total and work-related CVDs (WRCVDs) rates stratified by age and year and explore the relative contributions of work to the CVD risk. *Methods*: This study was conducted using two populations from the Labor Insurance Database as the working population and the National Health Insurance Research Database as the general population. We included all people aged 15–75 years from 2006 to 2013. All CVD events and WRCVD events were identified. A Poisson regression was used to estimate the morbidity rate ratio (RR) stratified by age and period, and an RR adjusted for residual confounding was also used. *Results*: Incident CVD rates increased with aging in the general population (from 1113.55 to 1853.32 per 100,000 persons), and WRCVD rates increased in the working population over time (from 2.10 in 2006 to 8.60 in 2013 per 100,000 persons). In the age and period analysis, CVD attacks showed disparities in different populations. The RR of the WRCVD risk was mainly in the working population aged >45 years, and the RR of the CVD risk occurred in the oldest group (aged 55–64 years) of the general population. The population-attributable risk of working exposure was 13.5%. After eliminating residual confounding factors, higher population attributed risk (PAR) work-related excessive CVD risk mainly occurred in workers aged 25–34 and 35–44 years. A decreasing PAR trend was found in the age groups as follows: 15–24, 25–34, 35–44, 45–54, and 55–64 years, with percentages of 17.64%, 16.89%, 16.46%, 10.6%, and 0.65%, respectively. *Conclusions*: There is evidence that period and age trends of CVD rates differed between the working population and general population. Relative effects attributed to work were more severe in younger workers, particularly in workers aged <55 years.

## 1. Introduction

In recent years, work overload, inducing cerebrovascular and cardiovascular diseases (CVDs), has become a global epidemic issue [1,2]. Globalization has fostered socioeconomic changes, demographic transitions, and rapid industrialization, leading to various occupational classes suffering from attacks of CVDs [3,4,5,6]. The annual number of CVD-related deaths is projected to increase from 17 million in 2008 to 25 million by 2030 [7]. Working populations represent 50% of total CVD deaths, and at least 25% of work disability is related to CVDs [1,5]. The global burden of mortality from work-related diseases may be as high as 5.2 million [8,9].

Risk factors for CVDs in workers include age, occupation type, lifestyle, and behavioral and social determinants. Previously, causal relationships with CVDs were found for work stress [10], long working hours causing work overload [11], job insecurity [12], and physicochemical factors [1]. Numerous studies have suggested that a macro-level of the sociopolitical context influences occupational diseases [13,14,15].

In Taiwan, the government recognizes CVD attacks caused by overwork as work-related (WR)-CVDs [16]. An occupational disease record and compensation system was established by the Taiwanese government. The Ministry of Labor in Taiwan produced diagnostic guidelines for occupational CVDs that were first promulgated in 1991, and guidelines for work-related CVD (WDCVD) criteria were revised in 2004 and 2010 [17].

There is a growing evidence of a causal relationship between work stress and CVD incidence [16], but there is less evidence of the contribution to the macro dimension of occupational CVDs. While the revised guidelines were able to more correctly guarantee a WRCVD declaration, they were insufficient in providing the relative contribution of work to the risk of CVDs. Therefore, we assessed the annual age-specific WRCVD rates in the working population as well as CVD attack rates in the general population from 2006 to 2013. We also attempted to explore possible impacts of CVD risks and the relative contributions of age and working year.

## 2. Methods

### 2.1. Data Sources

This study was conducted using the Labor Insurance Database (LID) for the working population and the National Health Insurance (NHI) Research Database (NHIRD) for the general population. Historical records of occurrences of occupational accidents, diseases, and death events in the working population are compiled by the Bureau of Labor Insurance, Ministry of Labor [17]. All work-related information (such as work-related attacks) for all employees in Taiwan is included in the LID. Approximately 99% of people in Taiwan participate in the NHI program. The Longitudinal Health Insurance Database 2005 (LHID2005), a subset of the NHIRD database, was released by the National Health Research Institute from the Ministry of Health and Welfare [18]. The LHID2005 contains claims data of 1,000,000 beneficiaries randomly selected from the Registry of Beneficiaries of the NHIRD in 2005. This database contains registration files and original claims data for reimbursement.

### 2.2. Study Populations and Case Ascertainment

We included all populations aged 15–75 years from 2006 to 2013. Only subjects with missing age or year were excluded from the populations (total < 0.3% every year). In the working population, WRCVD events were identified according to records of registered work-related CVD accidents and deaths. The denominator is based on the number of insured working persons of that age and period. There were 8,681,139 participants in 2006, 8,799,404 participants in 2007, 8,795,243 participants in 2008, 9,029,277 participants in 2009, 9,397,603 participants in 2010, 9,725,755 participants in 2011, 9,709,501 participants in 2012, and 9,745,793 participants in 2013. In the general population, participants discharged with a related diagnosis from inpatient visits, outpatient visits, or deaths were defined as CVD event cases. Onset time was set as the first date of having a CVD diagnosis. Patients with newly diagnosed CVDs were identified as patients who had at least two ambulatory visits over 3 months or one inpatient visit. All registrants of that age and period were included as the denominator of the general population. There were 679,831 persons in 2006, 696,554 persons in 2007, 712,656 persons in 2008, 736,068 persons in 2009, 757,013 persons in 2010, 768,543 persons in 2011, 774,429 persons in 2012, and 780,150 persons in 2013.

### 2.3. Definition of WRCVDs and CVDs

In the working population, the guidelines recognized as WRCVD injury or death events included: (1) cerebrovascular diseases (cerebral hemorrhage, cerebral infarction, subarachnoid hemorrhage, and brain damage caused by severe hypertension), and (2) heart diseases (myocardial infarction, acute heart failure, dissecting aneurysm of the aorta, angina pectoris, serious cardiac arrhythmia, cardiac arrest, and sudden cardiac death). In the general population, CVDs were identified according to claims data. According to the ninth revision of the International Classification of Diseases (ICD-9), WRCVD and CVD codes were identified as follows—acute myocardial infarction: 410; congestive heart failure: 428; dissection of the aorta: 441; cerebral thrombosis with cerebral infarction: 434.01; cerebral embolism with cerebral infarction: 434.11; subarachnoid hemorrhage: 430; intracerebral hemorrhage: 431; and hypertensive encephalopathy: 437.2. Ultimately, 408 observed WRCVD events from the working population and 109,236 observed CVD events from the general population were found.

### 2.4. Statistical Analysis

The LID and NHIRD are supervised separately by two departments, and they could not be linked at an individual level. Grouped data of CVD or WRCVD and related population size by age and year were extracted from the databases. These participants were separated into six age categories: 15–24, 25–34, 35–44, 45–54, 55–64, and ≥65 years, and these were calculated every calendar year during 2006~2013. Crude annual age-specific CVD morbidity (first-ever-in-a-lifetime event) and/or attack rates (all events, including recurrent events) with 95% confidence intervals (CIs) per 100,000 persons were calculated. A generalized estimation equation (GEE) with a log link and Poisson assumption (as a log-linear model) were used. The model with CVD or WRCVD events as the dependent variable and population size as the offset was conducted using pooled technology.

Insurance payment and occupational disease registration rules varied with age and year during the study period. Two sets of models for each age group and each period were separately used: models adjusted for age and period, and age–period models additionally adjusted for residual confounding.

The residual adjustments were made for two reasons. First, the LHID2005 is a subset database of the whole population, and the sampling fractions in each age and period are unknown. Second, the background exposure of the general population could not be separated from the working population. This method is common in the analysis of vaccine population vs. total population [19,20], such as in the example presented by Vamos et al. [21]. Therefore, individuals who were and those who were not in the working population should have similar CVD risks after background adjustment, with an expected morbidity rate ratio (RR) of 1.0 for the general population. The effect estimates of risk in the general population were used to adjust for the residual confounding that occurred in the working population as the adjusted RR (RR_adjusted_).

RR_adjusted_ = Exp(β _working pop_ − β _general pop_)

To calculate 95% CIs for the RR_adjusted_, we resampled 500 times and 10,000 persons each time from the distribution of the observed populations in each age and period group. After taking the difference of each of the 500 sampled estimates, the 2.5th and 97.5th percentiles of the distribution were used to obtain 95% CIs for the adjusted RRs. The population-attributable risk (PAR) percentage for working was also calculated using the standardized rates as a supporting analysis. All statistical analyses were performed with SAS® v.9.3 software (SAS, Cary, NC, USA).

### 2.5. Ethics

Ethical approval was obtained from the Taipei Medical University-Joint Institutional Review Board (approval no.: TMU-JIRB N201510071).

## 3. Results

**Figure 1:**
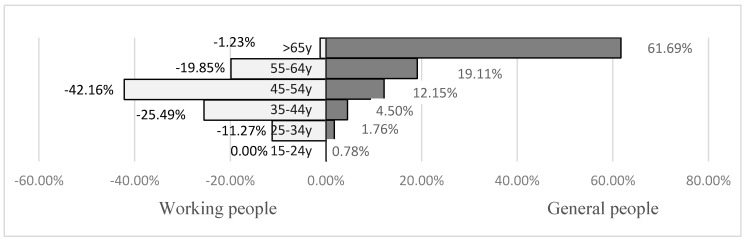
Comparisons of percentage of persons suffering from WRCVDs and CVDs in the working and general populations in Taiwan, 2006–2013, stratified by age. WRCVDs: work related cerebrovascular and cardiovascular diseases. CVDs: cerebrovascular and cardiovascular diseases. y: year.

Figure 1 and Table S1 show the percentages stratified by age among persons who suffered from CVDs in the working and general populations. WRCVDs had greater percentage contributions, than in the general population, among persons aged 45–54 (42.16%) and 35–44 years (25.49%) in the working population. In contrast, higher CVD attack rates were found in the older age group ≥65 years (61.69%) in the general population than in the working population.

Table 1 reports the number of event and annual age-specific CVD attack rates (per 100,000) in the working and general populations. In the working population, WRCVD attack rates increased approximately four-fold from 2006 to 2013 (2.10 to 8.60 per 100,000 persons). In the same interval, CVD rates in the general population slowly increased (1113.55 to 1853.32 per 100,000 persons) approximately two-fold. A significant age trend was shown in the general population, but such a trend was not seen in observations of WRCVDs.

**Figure 2:**
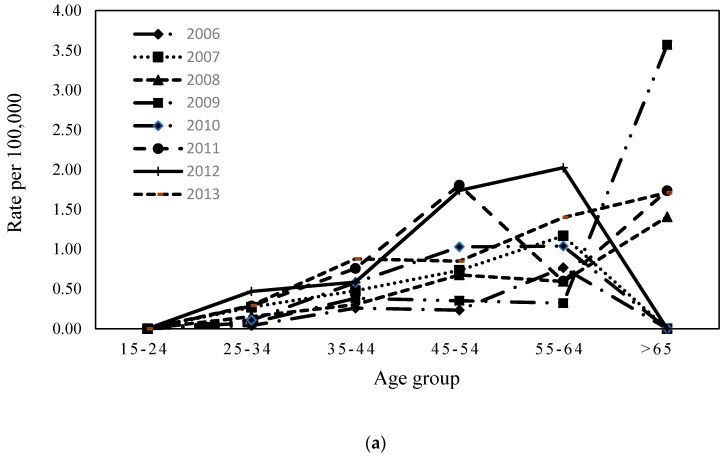

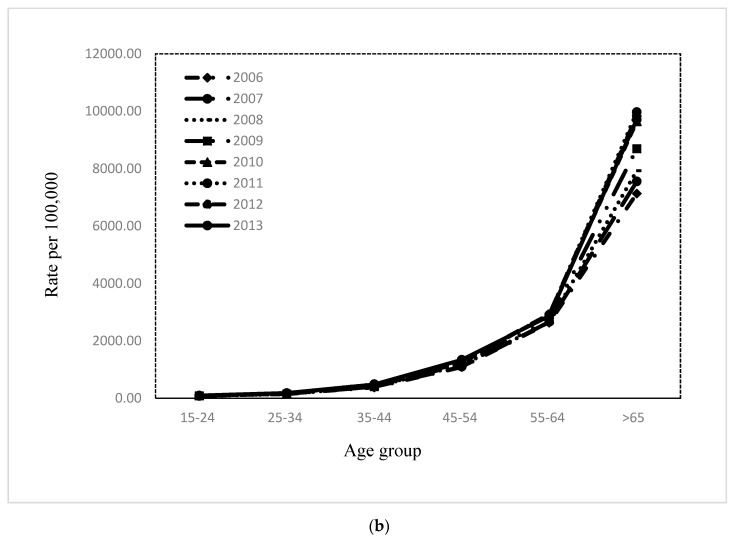
Trends of annual age-specific WRCVD rates and CVD rates (**a**) in the working population and (**b**) in the general population. WRCVDs: work related cerebrovascular and cardiovascular diseases. CVDs: cerebrovascular and cardiovascular diseases.

The trends of annual age-specific WRCVD attack rates and CVD attack rates are displayed in Figure 2a,b. WRCVD attack rates generally increased with age (shown in Figure 2a). Moreover, in the general population, a marked increase occurred in the age group ≥65 years, which had the highest CVD rates (as shown in Figure 2b).

An age–period model was used to estimate the RR and 95% CI for WRCVDs and CVDs, and results are shown in Table 2. The population aged ≥65 years was a very different population in the working population than in the general population. Therefore, persons aged ≥65 years old were excluded from the final analysis.

In the working population, period RRs were strongly significant in 2007 and 2010~2013 as compared to 2006 as the reference. An approximate four-fold increase in the WRCVD risk was indicated from 2006 to 2010–2013. Such significant effects of WRCVDs were also found in workers aged 25–64 years. The RRs for WRCVDs increased with age, but we found that the risk rose only two-fold. The highest risk (RR = 1.84; 95% CI = 1.37–2.46) in the 55–64-year age group was shown in comparison to that aged 35–44 years. In the general population, all RRs for CVD onset in each period and age group were significant.

In order to examine the evolution of workers suffering CVD risk disparities across periods and ages, we presented the RRs of CVDs in Table 2. For each concentration of work effect, adjusted RRs were calculated using RRs from the working and general populations. The adjusted RR slightly increased over time, with a considerable increase in 2012 (adjusted RR = 3.84; 95% CI: 2.38–5.61). Of note, there was an observed increase in the adjusted RR in the age group 25–34 years (adjusted RR = 1.08; 95% CI: 0.66–1.94), which significantly decreased in workers aged ≥45 years. At 55–64 years of age, contributions of the effect of work on the CVD risk were slight (adjusted RR = 0.29) but still strongly significant (95% CI: 0.20–0.37).

PARs contributed by working were also calculated using standardized rates. After age and year standardization, the PAR of working exposure was 13.45% (95% CI = 13.2–13.7%). There was a decreasing PAR trend in the age categories 15–24, 25–34, 35–44, 45–54, and 55–64 years, and the percentages were 17.64% (95% CI = 17.4–17.9%), 16.89% (95% CI = 16.7–17.1%), 16.46% (95% CI = 16.2–16.7%), 10.6% (95% CI = 10.4–10.8%) and 0.65% (95% CI = 0.58–0.69%) when standardized by year.

## 4. Discussion

As far as we know, this is the first longitudinal study to present the attributes of WRCVDs considered as occupational diseases by workers’ compensation systems for comparison to all CVDs in the general population. By comparing two population-based databases from national labor and health insurance, we found disparities between the working and general populations in CVD attack rates. A PAR of 13.45% was contributed from working exposure in populations aged 15–64. The contribution from occupations to CVD risk significantly decreased with age, from an RR_adjusted_ of 0.63 at 45–54 years to an RR_adjusted_ of 0.29 at 55–64 years, when compared to the reference group (persons aged 35–44 years) The PARs of working exposure also decreased with increasing age. This demonstrates that work and work-related factors are very significant risk factors in younger populations.

Three major findings were shown when we looked at temporal and age differences from the age–period analysis. First, WRCVDs and CVDs attacks increased with aging and period in populations aged 15–75 years from 2006 to 2013. However, their performances were different. Second, the magnitude of the differences in CVDs between the working and general populations substantially varied by age, with the largest disparities observed particularly among young and middle-aged adults in the main labor force. A pattern of adjusted RRs and PAR was shown, with significantly higher impacts focused on WRCVDs in young workers. In contrast, aging prominently led to increased CVD onset in the general population. Finally, disparities in period-specific adjusted RRs from CVDs for 2009 and 2013 were modest. However, they were all >1, indicating a substantial improvement in the reporting and registration of job-related CVDs after the revised guidelines were promulgated.

In our study, the age–period model was used to adjust for age and period influences [22], and RRs denoted the ratios of morbidity rates as a relative indicator for the reference group after adjustment. Taking account of background age and period trends, RR_adjusted_ was used to adjust residual confounding in the general population. In our observations, the annual CVD attack rates from 2006 to 2013 ranged from 1114 to ~1853 per 100,000. These findings are similar to those in developed countries including the United States, Australia, and Britain [23,24]. As far as we know, age is the strongest risk factor for CVDs in the population, and the aging of the population is projected to continue. We adjusted for the age effect in our age–period model analysis. In addition, the NHIRD population is a representative sample of the general population in Taiwan, which was good for excluding residual confounding.

In our analysis, the working population WRCVD attack rate increased with age. However, the age distribution of CVD events differed between the two populations. Among 109,236 CVD events in the general population, 61.69% were among people aged ≥65 years. However, among 408 WRCVDs, 42.16% were among workers 45–54 years old and 25.49% were among workers 35–44 years old. After eliminating background residual confounding, attributable work-related excessive CVD risk mainly occurred in workers aged 45–54 and 35–44 years. The effect was huge, i.e., fourfold (RR_adjusted_ from 0.29 at 55–64 years to 0.6–1.0 at 35–54 years). A Scottish study indicated that premature death from coronary heart disease remains a major contributor to the most affluent groups aged 35–44 and 45–54 years [25,26]. Since middle-aged workers in Japan have been reported to experience prolonged working hours and occupational stress related to CVD risk [27], much evidence has focused on associations between CVDs and occupational factors such as working hours and stress [28].

In a worldwide diagnosis of guidelines for occupational CVDs made in different periods, the governments of Taiwan, Japan, and Korea only recognize CVDs caused by overwork as WRCVDs. The Taiwan Labor Department published “Guidelines for the Diagnosis of Work-Related Cardiovascular Diseases” in 2004 and revised it to be more sensitive in December 2010. Our study showed the higher adjusted RRs of CVDs in 2007 and 2012. The WRCVD attack rate increased from 5.71 in 2010 to 8.60 in 2013 per 100,000 people in the post-2010 revised guideline period. Similarly, the Japanese government recognized WRCVD diagnostic criteria in late 2001, and a gradual increase was found in total compensated CVD occupational diseases [15]. Nevertheless, the Korean government produced occupational health standards in 2003 and found that compensated CVDs accounted for 26% of the total compensated diseases in 2003, which was a dramatic increase, but the proportion dropped to 7% by 2009 [1]. Reducing compensatory CVDs may be attributable to many preventive activities carried out by governments and employers, but occupational health policy advocacy may increase the annual recognition of WRCVDs. The possible directions need further study.

We provided an exploratory descriptive tool to examine the occupation-attributed relative risk by age and period by taking account of the residual confounding of unknown background factors. Most notably, the present study is the first to examine combined national labor and health insurance databases, which was ideal and strengthened the results. The CVD diagnosis was performed according to ICD-9 codes. WRCVD events were identified through the application and review of guidelines from the government, which identified occupational causes of acute circulatory diseases. The population size was representative and was based on national insurance databases of the workforce and general population. Therefore, the findings in this study are reasonable to present as epidemiological evidence.

Three limitations should be considered in this study. First, the application accuracy of occupational diseases is related to a willingness to recognize occupational causes of injuries or health problems. These assessments included personal exposures to environmental risk factors, for example, from evidence of job insecurity, working hours, job intensification, and management [29,30]. This might vary by age, period, social culture, and country. Problems are also related to the historical background of workers’ demands for protection and prevention or compensation and their employers seeking to deny or reduce their liability for work-related diseases and injuries. Second, CVD identification in the general population is based on ICD-9 codes. Due to data extraction limitations, all attack cases included recurring events. However, the stratum-specific rate ratio may have a lower impact in estimation because the extraction criteria for each group are the same. Finally, most WRCVD registered events are recorded for males. The estimations are unstable in some strata in separating by gender. This issue should be considered in the future.

## 5. Conclusions

CVDs occurred in different periods and age groups in the two insurance system databases. The relative effects attributed to work were more severe in the younger population. Persistent intensive assessment and management of overwork and preventing WRCVDs among young workers are important [31,32]. It should be noted that monitoring regimens across subgroups, as well as the most effective timing and efficacy of primary, secondary, and tertiary preventive interventions for public health policies should be determined in future studies.

## Figures and Tables

**Table 1:** Annual event number and age-specific attack rates (per 100,000) of WRCVDs and CVDs in working and general populations.

Age Group (Year)	2006	2007	2008	2009	2010	2011	2012	2013
Working population								
15–24	0	0	0	0	0	0	0	0
	0.00	0.00	0.00	0.00	0.00	0.00	0.00	0.00
25–34	1	7	4	2	3	8	13	8
	0.04	0.27	0.15	0.07	0.11	0.28	0.47	0.29
35–44	6	11	7	9	14	19	15	23
	0.26	0.48	0.30	0.38	0.58	0.76	0.58	0.88
45–54	5	16	15	8	24	43	41	20
	0.23	0.73	0.68	0.35	1.03	1.81	1.74	0.85
55–64	6	10	5	3	11	7	23	16
	0.77	1.17	0.59	0.32	1.04	0.60	2.02	1.40
≥65	0	0	1	2	0	1	0	1
	0.00	0.00	1.41	3.57	0.00	1.74	0.00	1.71
Total	18	44	32	24	52	78	92	68
	2.10	5.18	5.15	5.89	5.71	8.65	9.48	8.60
General population								
15–24	103	111	98	107	114	96	106	119
	82.07	90.21	81.24	87.59	93.22	77.67	85.06	94.91
25–34	225	223	215	222	241	282	254	262
	158.32	153.08	143.93	144.45	153.43	180.66	170.34	181.92
35–44	524	554	560	566	651	692	668	697
	376.66	396.79	403.33	404.20	462.34	488.88	466.95	483.13
45–54	1377	1426	1535	1623	1737	1818	1842	1919
	1086.85	1099.32	1154.03	1185.95	1241.42	1283	1290.35	1338.76
55–64	1853	2036	2180	2418	2812	3010	3243	3326
	2632.21	2660.95	2646.14	2725	2926.51	2906.89	2924.97	2866.08
≥65	5422	6193	7009	8223	9729	10,176	10,239	10,400
	7133.84	7554.28	7928.19	8691.93	9647.96	9970.51	9844.15	9710.91
Total	9504	10,543	11,597	13,159	15,282	16,074	16,352	16,723
	1113.55	1216.38	1318.42	1462.64	1670.51	1753.15	1798.21	1853.32

Event/attack rates (per 100,000) are shown. WRCVDs: work related cerebrovascular and cardiovascular diseases. CVDs: cerebrovascular and cardiovascular diseases.

**Table 2:** Morbidity rate ratios (RRs) and 95% confidence intervals (CIs) for WRCVDs and CVDs by age and period in the working and general populations.

	Working Population				General Population				RR adj †	95% CI of RR adj ‡
	Slope	RR	95% CI	*p* Value	Slope	RR	95% CI	*p* Value		
Period										
2006	Reference	1			Reference	1			1	
2007	0.87	2.39	(1.38–4.13)	0.0019	0.02	1.02	(0.97–1.06)	0.4407	2.35	(1.51–3.40)
2008	0.51	1.67	(0.93–2.98)	0.0839	0.03	1.03	(0.99–1.07)	0.2063	1.62	(1.06–2.50)
2009	0.13	1.14	(0.61–2.12)	0.6842	0.05	1.05	(1.01–1.10)	0.0127	1.08	(0.69–1.58)
2010	0.95	2.58	(1.51–4.40)	0.0005	0.12	1.13	(1.09–1.18)	<0.0001	2.28	(1.45–3.28)
2011	1.3	3.67	(2.20–6.13)	<0.0001	0.14	1.15	(1.11–1.20)	<0.0001	3.19	(2.18–4.66)
2012	1.48	4.41	(2.66–7.31)	<0.0001	0.14	1.15	(1.10–1.20)	<0.0001	3.84	(2.38–5.61)
2013	1.16	3.2	(1.90–5.38)	<0.0001	0.15	1.16	(1.11–1.20)	<0.0001	2.76	(1.85–3.93)
Age										
15–24	−23.98	0	(-)	0.9993	−1.62	0.2	(0.18–0.21)	<0.0001	0	
25–34	−0.92	0.4	(0.28–0.57)	<0.0001	−1.00	0.37	(0.35–0.39)	<0.0001	1.08	(0.66–1.94)
35–44	Reference	1			Reference	1			1	
45–54	0.57	1.76	(1.38–2.25)	<0.0001	1.02	2.78	(2.69–2.87)	<0.0001	0.63	(0.45–0.83)
55–64	0.61	1.84	(1.37–2.46)	<0.0001	1.85	6.35	(6.18–6.58)	<0.0001	0.29	(0.20–0.37)

RR adj †: exp (β labor insurance–β health insurance), 95% CI of RR. ‡: resampling by bootstrapping 500 times. The 2.5th and 97.5th percentiles of the distribution were used to obtain 95% CIs for the adjusted RR. -: unable to estimate. WRCVDs: work related cerebrovascular and cardiovascular diseases. CVDs: cerebrovascular and cardiovascular diseases.

## Supplementary Materials

The following are available online at https://www.mdpi.com/1660-4601/16/6/961/s1, Table S1: Percentage stratified by age and year among persons who suffered CVDs in the working and general populations.
