# Supplementary material for: Significant Impacts of Work-Related Cerebrovascular and Cardiovascular Diseases among Young Workers: A Nationwide Analysis
Source: Int J Environ Res Public Health. 2019 Mar 18;16(6):961. doi: 10.3390/ijerph16060961 (PMC6466191; doi:10.3390/ijerph16060961)
Supplement: Supplementary file 1 [file ijerph-16-00961-s001.pdf]

Supplementary Table S1. Percentage stratified by age and year among persons suffered WRCVDs and CVDs in working and general populations.

| Age year | Year of survey: Labor insurance n (%) 2006 | 2007 | 2008 | 2009 | 2010 | 2011 | 2012 | 2013 | Total |
|---|---|---|---|---|---|---|---|---|---|
| 15-24 | 0 ( 0.00) | 0 ( 0.00) | 0 ( 0.00) | 0 ( 0.00) | 0 ( 0.00) | 0 ( 0.00) | 0 ( 0.00) | 0 ( 0.00) | 0 ( 0.00) |
| 25-34 | 1 ( 5.56) | 7 ( 15.91) | 4 ( 12.50) | 2 ( 8.33) | 3 ( 5.77) | 8 ( 10.26) | 13 ( 14.13) | 8 ( 11.76) | 46 ( 11.27) |
| 35-44 | 6 (33.33) | 11 (25.00) | 7 ( 21.88 | 9 ( 37.50) | 14 ( 26.92) | 19 ( 24.36) | 15 ( 16.30) | 23 ( 33.82) | 104 ( 25.49) |
| 45-54 | 5 (27.78) | 16 (36.36) | 15 (46.88) | 8 ( 33.33) | 24 ( 46.15) | 43 ( 55.13) | 41 ( 44.57) | 20 ( 29.41) | 172 ( 42.16) |
| 55-64 | 6 (33.33) | 10 (22.73) | 5 (15.63) | 3 ( 12.50) | 11 ( 21.15) | 7 ( 8.97) | 23 ( 25.00) | 16 ( 23.53) | 81 ( 19.85) |
| >65 | 0 ( 0.00) | 0 ( 0.00) | 1 ( 3.13 | 2 ( 8.33) | 0 ( 0.00) | 1 ( 1.28) | 0 ( 0.00) | 1 ( 1.47) | 5 ( 1.23) |
| Total | 18 ( 4.41) | 44( 10.78) | 32 ( 7.84 | 24 ( 5.88) | 52 ( 12.75) | 78 ( 19.12 | 92 ( 22.55) | 68 ( 16.67) | 408 ( 100.00) |

| Age year | Year of survey: Health insurance n (%) 2006 | 2007 | 2008 | 2009 | 2010 | 2011 | 2012 | 2013 | Total |
|---|---|---|---|---|---|---|---|---|---|
| 15-24 | 103 ( 1.08) | 111 ( 1.05) | 98 (0.85) | 107 ( 0.81) | 114( 0.75) | 96 ( 0.60) | 106 ( 0.65) | 119 ( 0.71) | 854 ( 0.78) |
| 25-34 | 225 ( 2.37) | 223 ( 2.12) | 215 ( 1.85) | 222 ( 1.69) | 241 ( 1.58) | 282 ( 1.75) | 254 ( 1.55) | 262 ( 1.57) | 1924 ( 1.76) |
| 35-44 | 524 ( 5.51) | 554 ( 5.25) | 560 ( 4.83) | 566 ( 4.30) | 651 ( 4.26) | 692 ( 4.31) | 668 ( 4.09) | 697 ( 4.17) | 4912 ( 4.50) |
| 45-54 | 1377 (14.49) | 1426 (13.53) | 1535 (13.24) | 1623 ( 12.33) | 1737 ( 11.36) | 1818 ( 11.31) | 1842 ( 11.26) | 1919 ( 11.48) | 13277 (12.15) |
| 55-64 | 1853 (19.50) | 2036 (19.31) | 2180 (18.80) | 2418 ( 18.38) | 2812 ( 18.40) | 3010 ( 18.73) | 3243 ( 19.83) | 3326 ( 19.89) | 20878 (19.11) |
| >65 | 5422 (57.05) | 6193 (58.74) | 7009 (60.44) | 8223 ( 62.49) | 9729 ( 63.65) | 10176 ( 63.31) | 10239 ( 62.62) | 10400 ( 62.19) | 67391 (61.69) |
| Total | 9504 ( 8.70) | 10543 ( 9.65) | 11597 (10.62) | 13159 (12.05) | 15284 ( 13.99) | 16074( 14.71) | 16352 ( 14.97) | 16723 ( 15.31) | 109236 (100.00) |

WRCVDs: work related cerebrovascular and cardiovascular diseases. CVDs: cerebrovascular and cardiovascular diseases.
